# Vestibular disorders in elderly patients: characteristics, causes and consequences

**DOI:** 10.11604/pamj.2014.19.146.3146

**Published:** 2014-10-15

**Authors:** Olusola Ayodele Sogebi, Adekunle Joseph Ariba, Taibat Olusola Otulana, Bamidele Sanya Osalusi

**Affiliations:** 1 ENT Unit, Department of Surgery, College of Health Sciences, Olabisi Onabanjo University Teaching Hospital (OOUTH), Sagamu, Nigeria

**Keywords:** Dizziness, elderly, vertigo, causes, characteristics, Nigeria

## Abstract

**Introduction:**

This study assessed vestibular disorders in elderly patients, describing the causes, clinical characteristics, therapies and treatment outcomes.

**Methods:**

Five-year hospital-based prospective study, conducted at the ENT clinic of a tertiary referral center. Subjects were consecutive elderly patients with dizziness, treated and followed-up for a minimum of six months. Data was generated using structured questionnaire and case record files. Analyzed results were presented in simple descriptive forms as graphs and tables.

**Results:**

Among the elderly patients, prevalence of vestibular disorders was 18.6%, 49.1% were retired, 71.9% were married, M:F was 1:1.1. Mean age ±SD were 69.4±1.1 and 69.0±0.8 years for males and females respectively. 56.9% of the patients presented early on experiencing the vestibular symptoms. The symptoms were associated with nausea or vomiting in 26.3%, with an aura in 12.3%. While 50.9% of the patients experienced intermittent symptoms, laterality of the symptoms was not clear in 45.6%. Positional vertigo was diagnosed in 33.3% while in 17.5%, the symptoms could be attributable to previous trauma or assaults. 31.6% of the elderly were referred to ENT surgeons by other specialties, 45.6% were managed with multidisciplinary approach, while 82.5% had the vestibular symptoms initially controlled with labyrinthine sedatives. At follow-up, 43.9% had intermittent periods of recurrence of symptoms.

**Conclusion:**

Prevalence of vestibular disorders in elderly patients is high, most patients present early with intermittent, relatively innocuous symptoms which may be difficult to lateralize. Positional vertigo was the most common cause, it is frequently relieved with labyrinthine sedatives but tends to recur intermittently.

## Introduction

Primary care clinicians, otolaryngologists, ophthalmologists and neurologists are sometimes confronted with patients who complain about inability to maintain their balance. Balancing is an interplay and integration of contributions from vision, vestibular sense working in conjunction with the cerebellum, proprioception, muscle strength and reaction time [1]. Dizziness is a general term for a sense of imbalance or disequilibrium and it affects approximately 20% to 30% of the general population [2]. The most common causes of dizziness are peripheral vestibular disorders, but physicians must differentiate between complaint of dizziness that is non-specific ranging from disequilibrium, presyncope, lightheadedness, giddiness, fainting attacks, to central nervous system disorders [2, 3]. Vertigo is a subtype of dizziness and refers to an erroneous perception of self or object motion or an unpleasant distortion of static gravitational orientation as a result of a mismatch between vestibular, visual, and somatosensory systems [4].

It was reported that about two-third of people with dizziness may have a vestibular etiologic diagnosis [5]. Dizziness often arises from malfunctioning of the vestibular apparatus in the ears (peripheral) and its connections to the central nervous system (central). Thus in the general population, it can be associated with diseases of the vestibular labyrinthine resulting from head injuries, infections, other types of assaults in the ear, as well as drug-induced cytopathic changes. With advancing age, there is a gradual but progressive loss of functioning of the vestibular sensory cells and this can manifest as dizziness.

The effects of dizziness in the elderly can be particularly disturbing, as it has been associated with depressive symptoms, perceived fatigue, excessive drowsiness, recurring falls and fall-related injuries such as fractures of long bones [1, 6]. Thus there is a growing public health concern about dizziness and balance disorders among the elderly worldwide. The prevalence of dizziness and vestibular disorders among elderly subjects vary from different locations. This may be due to non-uniformity in terminology, the criteria used for estimating the balance disorder, as well as disparity in the age regarded as elderly. The complaint of dizziness was reported by 45% of community dwelling elderly Brazilians [6], while in Columbia it was 15.2 percent [7]. In Gothenburg, Sweden, the overall prevalence of balance problems at age 70 was 36% in women and 29% in men while rotatory symptoms occurred between 2 and 17% of the elderly [8]. In Massachusetts, USA approximately one in five (19.6%) elderly (>65years) persons experienced annual problems with dizziness or balance, of which vertigo constituted 30.1 percent [9]. In a study on ageing in Ibadan, Nigeria, a prevalence of dizziness among community dwelling elderly (>65years) subjects was 24.8 percent [10].

Few studies have focused on the etiology of vertigo, specific peripheral diseases especially Meniere's disease, cervical vertigo, audiological and vestibular tests reports among adult population in sub-saharan Africa, including Nigeria. However, little is known about the effects of vestibular disorders in the elderly population, therapies commonly prescribed for controlling these disorders, and outcome of the treatments. These may not be unconnected with difficulties in follow-up and high default rate among our patients especially when they improve clinically. This study aimed to explore the clinical characteristics, causes, consequences and the outcome of treatment among elderly patients with vestibular disorders. It will assist in policy formulation for improved health and general well-being among elderly people, as well as appraise the efficacy or otherwise of our treatment modalities, and propose if there is a need for modification.

## Methods

Study design: This was a hospital-based prospective study. The study protocol was approved by the OOUTH- Health research and ethics committee HREC. Setting/study location: The study was conducted in the department of Ear, Nose and Throat (ENT) of Olabisi Onabanjo University Teaching Hospital (OOUTH), Sagamu, Nigeria-a tertiary referral center. The sources of these patients were referrals from the departments of family medicine (general out-patient department), internal medicine, ophthalmology, and from general practitioners in and around Sagamu.

Sampling criteria/technique: Participants were consecutive patients aged sixty years and above (operationally defined as elderly). Before seeking consent, patients were informed about the nature, general criteria for inclusion, benefits, and the fact that the patients may voluntarily decline to continue participation in the course of study without affecting their treatments. The main recruitment question was whether the patient experienced dizziness defined as the sensation of the patient turning around relative to his/her environment, or vice-versa. Patients that consented were included in the study, and those who did not consent were excluded. Also excluded were patients with other forms of dizziness other than as described above, patients with severe hearing impairment with associated difficulties in communication, those that were too weak to undergo vestibular assessments, patients with active ear infections, and those with tympanic membrane perforations.

### Study period: the study was conducted between may 2008 and december 2012

Data sources and collection procedure/technique: Information gathered from the patients by directly applying a structured questionnaire to each of them, and also from the case record files to generate data. The information obtained included socio-demographic data such as age, sex, occupation and marital status. The main distinguishing question was whether the patient experienced a sensation of turning around relative to the environment, or vice-versa. An affirmative answer to this question led to the characterization of the dizziness, in terms of onset, and duration of symptoms, associated symptoms of nausea and vomiting, premonition in the form of an aura, the behavior of the dizziness, aggravating factors and its laterality noted on neurovestibular clinical examination. Previous medical consultations, treatment as well as the source of referrals were also noted.

Neurovestibular examination was conducted on each of the patients, and particularly clinical tests for balance, including static balance test *(Rhomberg's) test, Unterberger test and dynamic balance test, tandem walk. Dix-Hallpike test* was performed on each of the patients with suspected positional vertigo. The clinical diagnosis, investigations, and the mainstay of managing the dizziness were recorded. All the patients were followed up for at least six months post initial presentation, and the outcome of treatment of the vestibular disorders was noted.

### Data analysis

The results were analyzed using SPSS version 19.0 (Illinois, USA), and presented in simple descriptive forms as graphs and tables. Discrete variables were described with proportions while continuous variables were described with measures of central tendencies such as means, and of dispersions as standard deviations.

## Results

**Figure 1:**
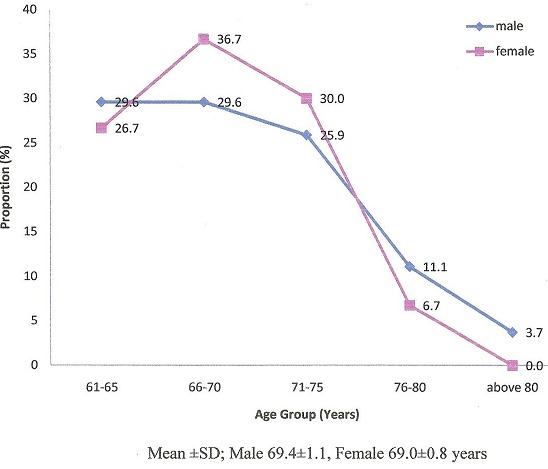
Age distribution according to sex of patients

There were sixty six elderly patients with vestibular disorders seen during the study period, constituting a prevalence of 18.6% among the total number of three hundred and fifty-four elderly patients managed during this period. Among these patients, fifty seven participated in the study with almost half of the subjects (28/57 = 49.1%) being retired, while 41/57 = 71.9% were married. There were 27 males and 30 female patients, M:F = 1:1.1. The age ranged between 61 and 81 years, Mean±SD were 69.4±1.1 and 69.0±0.8 years for males and females respectively. The age distribution according to the sex of the patients is shown in Figure 1.

Table 1 shows the characteristics of the vestibular disorders in the patients. The median duration of symptoms before presentation was 3.0weeks, which was used as the dividing line into short or long durations of symptoms; 56.9% of the patients presented within three weeks of experiencing the vestibular symptoms. The vestibular symptoms were associated with nausea or vomiting in about a quarter (26.3%) and with the experience of an aura in 12.3% of the patients. Half of the patients (50.9%) experienced intermittent symptoms and the laterality of the symptoms was not clear in 45.6% of the patients.

**Table 1:** Characteristics of vestibular disorders in elderly patients

Variable	n	%
Duration of symptoms		
Short ≤3 weeks	34	59.6
Long > 3 weeks	23	40.4
Mean ±SD	4.04±2.7	
**Associated with nausea or vomiting**		
No	42	73.7
Yes	15	26.3
Character		
Aura absent	50	87.7
Aura present	7	12.3
**Behavior**		
Transient	20	35.1
Intermittent	29	50.9
Continuous	8	14.0
**Laterality**		
Unilateral	10	17.5
Bilateral	21	36.8
Not clear	26	45.6

The clinical spectrum of the causes were varied, however a third (33.3%) of the patients experienced positional vertigo, while the cause of the vestibular disturbance could not be ascertained in 19.3% of the patients. In 17.5%, the vestibular symptoms could be attributable to various forms of previous trauma or assaults like head injuries, or drugs ingestion. The detail of the distribution is shown in Table 2.

**Table 2:** Clinical spectrum of vestibular disorders

Diagnosis	n	%
Positional vertigo	19	33.3
Meniere's disease	5	8.8
Vestibular schwannoma	1	1.8
Post trauma	10	17.5
Cerumen impaction	4	7.0
Cervical vertigo	7	12.3
Unknown	11	19.3

The clinical course of the patients also revealed variability as depicted in Table 3. Majority of the patients (31.6%) were referred to the ENT surgeons by general duty and family physicians, while 28.1% attended the ENT clinic without any formal referral. The other referrals were from physicians especially neurologists, ophthalmologists, and other specialties. 54.4% of the patients were managed by the ENT surgeon alone, while others were co-managed with other disciplines including neurologists, ophthalmologists, orthopedic surgeons and physiotherapists. Majority of the patients (82.5%) had the vestibular symptoms initially controlled with labyrinthine sedatives, while a tenth (10.5%) were controlled mainly with diuretics, and others with other modalities like other sedatives, tranquilizers, and ear syringing was employed in 7.0% of the patients.

**Table 3:** Clinical course of the patients

Parameter	n	%
**Referral source**		
Nil	16	28.1
Family/General duty physician	18	31.6
Ophthalmologist	7	12.3
Internal physician	11	19.3
Others (General surgeons, etc)	5	8.8
Management discipline		
Single (ENT alone)	31	54.4
Multiple (ENT and others)	26	45.6
Main therapy		
Labyrinthine sedatives	47	82.5
Diuretics	6	10.5
Others	4	7.0
**Outcome of treatment (at ≥6/12 post initial management)**		
Improved, no recurrence	22	38.6
Improved with recurrence	25	43.9
Not improved, worse	3	5.3
Referred to other centers	2	3.5
Indeterminate	5	8.8

While some patients were followed-up to close to three years, the minimum period of follow-up for each patient was six months after the initial consultation and management. The outcome from the follow-up revealed that 38.6% of the patients had significant abatement of symptoms without recurrence, while 43.9% had intermittent periods of recurrence of symptoms. 5.3% of the patients either did not improve or had worsening of the symptoms.

## Discussion

The vestibular system provides information to the central nervous system (CNS) on the orientation of the body in space, together with somatosensorial information. Thus, when the vestibular system is compromised by aging, through progressive degeneration and reduction in the number of labyrinthine hair cells and ves¬tibular receptor ganglion cells, the CNS will experience difficulties in dealing with reduced or conflicting sensorial information [11]. The most common manifestation of vestibular disorder displayed in general population was spinning sensation [12]. The prevalence of 18.6% noted in this study depicts that almost one in every five elderly patients that presented in our ENT clinic had vestibular disturbances similar to that reported in Boston, USA [13]. This is higher than the prevalence of between 2.0 and 17.0% reported in other developed countries [7, 8] but lower than that reported in the Ibadan study on ageing [10]. The difference may be attributable to the hospital-based nature of our study, and the fact that some elderly patients in the community are incapacitated and find it difficult to access medical care from hospitals.

The vestibular symptoms can be disturbing and with increasing severity or recurrence, may even force subjects to premature retirement from work; almost half of the patients in this study were retired. Majority (71.9%) of our patients remained married possibly because they were relatively young elderly with average age of 69 years. The apparent equality in frequencies of vestibular disease between the sexes with M:F of 1:1.1, was at variance with reports from other studies which reported a female preponderance [12]. This may be a reflection of the socio-demographics of the elderly in our communities. Stevens et al, [14] however noted that dizziness problems were not associated with gender. Many of our elderly subjects for reasons of believe, culture, ignorance and possibly economy, still patronize unorthodox medical practitioners. However the fact that 59.6% of the subjects presented within 3weeks, (mean was 4.0 weeks) may lend credence to the severity and perhaps the frustration associated with difficulty in controlling the symptoms at previous facilities. In addition, there was associated nausea or vomiting in 26.3% of the subjects complicating the distress. The aura can be a warning signal of an imminent vertiginous episode, and the subject can take remedial measures to prevent or possibly ameliorate its effects. However, only 12.3% of our patients affirmed the presence of an aura.

Half of our patients (50.9%) had intermittent symptoms, while 14.0% had continuous episodes lasting for several minutes and occasionally lasting over one hour. While prolonged dizziness may be one of the cardinal symptoms suggestive of CNS disorders [15], it sometimes results from continued irritation and provocation of the vestibular apparatus. The peripheral vestibular pathology is often asymmetric and may be difficult to lateralize. Methods of lateralizing of vestibular lesions by alternate binaural bithermal caloric test (ABBT) with its various modifications, electronystagmography, and the more modern methods [16] are not available in our center, thus it was difficult to lateralize the symptom in 45.6% of our patients. It is obvious that the typical characteristics of vestibular disturbances experienced by our elderly patients were of short duration, mostly without an aura, intermittent, probably non-lateralizing and not associated with nausea and vomiting.

These characteristics may suggest more of peripheral vestibular lesions of less serious magnitude. The most common diagnosis made in our patients was positional vertigo. This was underscored by that fact that in 49.2% of the patients, the symptoms were either provoked or aggravated by turning the neck, head or changes in the position of the body. Many authors [3, 17, 18] had reported benign paroxysmal positional vertigo (BPPV) as the most common type of vestibular disorders found in adults and elderly patients. BPPV is a diagnosis with stringent criteria, including intermittent positional vertigo lasting less than one minute, and confirmation with Dix-hallpike test [5]. Some of our patients, despite having positional vertigo, did not completely fit into the above criteria. Fife [17] however believes that these other forms of positional vertigo are variants of BPPV. While positional vertigo can be benign and amenable to treatment with canalith positioning technique like Epley and Semont maneuvers, the paroxysmal recurrences in BPPV can become troublesome.

In almost a fifth (19.3%) of our elderly patients, the cause of the vestibular disturbances was not known. Post and Dickerson [3] also noted that a final diagnosis was not obtained in about 20 percent of their patients with dizziness. Delineating a vestibular pathology can sometimes be a daunting task, as it will involve careful and meticulous clinical oto-neurological evaluation, and sometimes specific investigations. These investigations are not only expensive, but mostly uncomfortable for the patients. This is due to their provocation of vertiginous attacks that the patient is even afraid of, thus compounding the situation in elderly subjects. Thus the physician in a developing country is hampered technically in exploring a diagnosis by unwilling and possibly incapable patients, scarce and expensive investigations, and thus confined to clinical evaluation most of the time. A working knowledge of these disorders will help the physician make the diagnosis efficiently by gathering key elements of the history and fine-tuning diagnostic testing [19].

Vestibular disturbances secondary to assaults and injuries to the labyrinth seems to be common in our environment, being attributable to 17.5% of the causes in this study. Falls are the leading cause of traumatic brain injuries (TBI) for older adults [20–22], displacing road traffic accidents, which occurs in the general adult population [23]. Conversely, vertiginous episode in the elderly also predispose them to falls and other domestic accidents including head injuries [1]. Labyrinthine assaults also occur from ingestion of medications; these include across the counter drugs like acetylsalicylate, which many elderly patients take for head and body aches, or a part of prescription for other ailments especially among hypertensives.

The pathophysiology of cervical vertigo has been attributed to disorders of the cervical spine, the vertebral artery and the cardiovascular system [24] which are common in the elderly. We also discovered vertigo caused by cerumen impaction in 7.0% of our patients. Cerumen impaction has been reported to be disproportionately common among elderly patients compared to others in the population. While symptoms are usually reduced hearing and sometimes otalgia, vertiginous episodes may also occur [25]. In these patients, the vestibular symptoms completely abated with the management of the impacted wax with softening and subsequent ear syringing. While majority of our patients had peripheral causes of vestibular disturbance similar to other studies [26], a diagnosis of possible intracranial neoplasm in one patient could not be confirmed, before referral to a neurosurgeon. However the other symptoms of headache, diplopia, and dysarthria complained by the patient were suggestive.

The symptoms of vestibular disorders can be confusing, thus patients tend to present to different doctors. In this study, about a third (31.6%) of our patients were formally referred to otolaryngologists by general duty and family physicians, similar to the pattern observed in the USA [13]. This underscores our structured health system expressing some decorum regarding the referral system. It can also be attributable to our center being a university teaching hospital where most units will accept patients mainly on referral. Regardless of this however, 28.1% of these patients were not referred.

The principles of management of vestibular disorders include initial evaluation, control of vestibular symptoms, re-evaluation, treatment and prevention of recurrence. After the initial evaluation, the vestibular symptoms are controlled with medications including labyrinthine sedatives, generalized central nervous system sedatives or tranquilizers. However, there is no single effective medication for vertigo and in clinical practice a combination of drugs are used, including antihistamines and anti-emetics [27]. While acutely administered anti-vertiginous medications can be given to control an attack, they have limited benefit in patients whose episodes last only a few seconds. Most of our patients (82.5%) had remission and control mainly with labyrinthine sedatives. Management of vestibular disorders however surpasses just the control of symptoms, necessitating a re-evaluation to ascertain cause and institute appropriate treatment. This often entails a multidisciplinary management, incorporating other treatment modalities like vestibular habituation, physiotherapy, psychotherapy and possibly cognitive-behavioral therapy [28]. Close to half (45.6%) of the elderly patients managed in this study had multi-disciplinary care. In addition, multidisciplinary care may be necessary in these patients due to presence of concomitant diseases requiring specialists’ attention.

The essence of care for the elderly pivots upon achievement of good quality of life, especially regarding the vestibular symptoms. The outcome of treatments in our patients after a minimum of six-month follow-up period after the initial management revealed that 38.6% of the patients had complete resolutions, but almost half had relatively unsatisfactory outcomes comprising 43.9% with recurrences and 5.3% with worsening symptoms. This outcome may be attributable to two main factors, namely inadequate diagnostic equipment with resultant inadequate characterization, and patient factors. Many patients actually defaulted treatment regimen for reasons ranging from attending many specialized clinics, different modes of operations in different clinics, inadequate explanation to lack of empathy by medical personnel. Individual patient limitations from frailty of the body and other personal social problems may also have compounded the default rate. There may be a need to modify our treatment approach and modalities especially regarding management of elderly patients.

It is an established fact that despite the relatively poor health systems in the developing countries, the population of the elderly is on the increase [29]. The elderly should be regarded as a peculiar population with unique characteristics and needs, thus geriatric care units should be made available in our tertiary health institutions, as is currently the case in many developed countries. This should take care of the elderly holistically and will thus reduce exhaustion and frustration from the stress of interdepartmental/units referral protocols. There should be a commensurate increase in quality of care, to achieve an improved quality of life. Adequate medical personnel and nursing staff to which individual patients can complain, share their fears and concerns are also needed for care to be effective. Communication is of essence in the elderly [30, 31] and these should be taken seriously. Some of the patients in this study were actually followed-up through telephone communications and were not re-evaluated clinically, thus limiting the validity of follow-up outcomes.

The other limitations that were observed in this study included the insufficient characterization of the vestibular disorders resulting from lack of necessary equipment, the hospital-based nature of the study which may not satisfactorily reflect what obtains in the community, and the fact that some patients were lost on follow-up. Despite these however, this descriptive study has been able to characterize the pattern, behavior and outcome for vestibular disorders in our elderly patients. There will be a need for replicating this study at the community level, and also conduct a controlled and comparative study of these disorders between the elderly and other population groups.

## Conclusion

In conclusion, almost one in every five elderly patient had experienced vestibular disorder which presented early with intermittent, relatively innocuous symptoms and may be difficult to lateralize. While positional vertigo was the most common cause, it is frequently relieved with labyrinthine sedatives but tends to recur. The need for specialized comprehensive geriatric care and practice were emphasized.
